# A Case of Group A Streptococcus Bacteremia and Infective Endocarditis Caused by Right Ovarian Tube Endometriosis Where the Patient’s Perspective Was Key to the Diagnostic Process

**DOI:** 10.7759/cureus.50081

**Published:** 2023-12-06

**Authors:** Arisa Ito, Taiju Miyagami, Mayu Suzuki, Tsubasa Nishina, Toshio Naito

**Affiliations:** 1 Department of General Medicine, Juntendo University Faculty of Medicine, Tokyo, JPN; 2 General Medicine, Jyuntendo University Hospital, Tokyo, JPN

**Keywords:** bacteremia, abdominal pain, vaginal system, infective endocarditis, group a streptococcus pyogenes

## Abstract

*Group A streptococcus pyogenes *(GAS) is a common organism that can cause upper respiratory infections. We encountered a case where GAS caused infective endocarditis (IE) due to an entry from the vagina. In this case, although echocardiography was negative, we were able to make a diagnosis of IE based on the 2023 Duke International Society for Cardiovascular Infectious Diseases Criteria, and we started antimicrobial therapy for IE. However, the patient subsequently developed persistent abdominal pain, which was atypical; hence, we reviewed the differential diagnosis. It is difficult to locate the primary site of infection because GAS rarely causes vaginal infections, and vaginal infections rarely cause IE. This case highlights the significance of revisiting medical history and the value of using a system 3 approach to refine diagnostic directions.

## Introduction

In humans, Group A Streptococcus pyogenes (GAS) is an indigenous bacterium of the skin and oral mucous membranes that commonly causes upper respiratory infections. This can often result in a clinical diagnosis of pharyngitis or tonsillitis, most common in children and adolescents (accounting for 15%-30% of such infections). GAS can progress to critical infections such as peritonsillar abscesses or systemic invasive infections [1]. It can also result in post-infection sequelae, including acute glomerulonephritis, acute rheumatic fever (ARF), and rheumatic heart disease (RHD) [2]. Systemic GAS infection can be fatal, as it is estimated to be responsible for over 163,000 deaths annually [3] and is postulated to be the fifth most lethal pathogen worldwide [4].

Infective endocarditis (IE) is a systemic septic disease in which bacterial aggregation on the heart valves causes cardiac damage, and bacterial clumps on the valves disseminate throughout the body causing a variety of clinical manifestations, including bacteremia and vascular embolism [5]. One recent study found that the incidence of IE in the United States increased over 10 years [6]. However, the precise incidence of IE is challenging to determine, owing to updates in clinical definitions over time and changes in the prevalence rates of predisposing factors [7]. Streptococcal infections, excluding Streptococcus gallolyticus, account for 5% of IE cases [8]. It is essential to investigate the entry site of the infection in cases of IE to treat the original source of infection and prevent further disease recurrence. However, one report claimed that the initial entry site could not be determined in 26% of IE cases [2]. We encountered a case of IE caused by GAS-associated IE. While diagnosing the condition was relatively straightforward, tracing its origin proved significantly more challenging.

## Case presentation

A 46-year-old, otherwise fit and healthy woman presented to our hospital with symptoms of fever and watery diarrhea. She began experiencing a 40 °C fever and diarrhea, as well as abdominal pain and hematuria, one week prior. The pain was located in the lower abdomen and had a dull character, with a severity of 5/10 on a subjective pain scale. She also reported left-side chest pain that was exacerbated by inhalation. She had no other upper respiratory symptoms. She worked in a kindergarten but had not had contact with anyone with an infectious disease. Her gestational history was gravida 2, para 2. She had a long-term partner but had not been sexually active for more than 10 years. An initial assessment at a local clinic prompted her referral to our facility.

On admission, the patient’s cognition was mildly impaired, with a Glasgow Coma Scale (GCS) score of E3V5M6. Her vital signs were as follows: pyretic, at 38 °C; tachycardic, with a heart rate of 113 beats/min; her blood pressure was 103/65 mmHg. Further, she had tachypnea with a respiratory rate of 32 times/min and an oxygen saturation of 96% when breathing regular room air. Our positive examination findings were Levine III/VI of a pansystolic murmur at the second intercostal space, immediately to the lateral left of the sternum. The patient also had left lateral abdominal tenderness, with no radiation or signs of peritonitis. There was a 1 cm non-tender erythematous lesion on the plantar aspect of her right foot. Otherwise, head and neck examinations were unremarkable.

Her symptoms of decreased cognition, fever, and tachypnea associated with a new cardiac murmur and peripheral erythematous lesion prompted us to suspect sepsis with IE, but a bedside transthoracic echocardiogram was inconclusive of cardiac valve vegetations. Laboratory tests revealed a white blood cell count of 39,500/μL (normal range: 3,500-9,000/μL; neutrophils: 95.2%) and a C-reactive protein level of 52.52 mg/dL (normal range: < 0.3 mg/dL). There was no decrease in platelet count or prolongation of prothrombin time, suggesting disseminated intravascular coagulation. Urinary tests revealed blood 3+ and a sedimentation test showed 10-19 red blood cells/high power field, with granular casts. Three sets of blood cultures were taken to be tested for suspected IE. Treatment with the broad-spectrum antibiotics meropenem (2 g q8h) and vancomycin (1 g q12h), covering gram-positive cocci, gram-negative rods, anaerobes, and methicillin-resistant Staphylococcus aureus, was started immediately, as per our usual protocol for sepsis. Contrast-enhanced computed tomography imaging from the chest to the legs showed no signs of deep vein thrombosis, pulmonary embolism, abscess, or space-occupying lesions. The lungs showed no inflammatory changes; however, a small left pleural effusion was observed. Some ascites were present on the pelvic floor with peritoneal thickening. The volumes of both pleural fluid and ascites were too small to aspirate. Transthoracic and transesophageal echocardiography did not show signs of vegetation. MRI of the head showed no obvious emboli or aneurysms.

**Figure 1:**
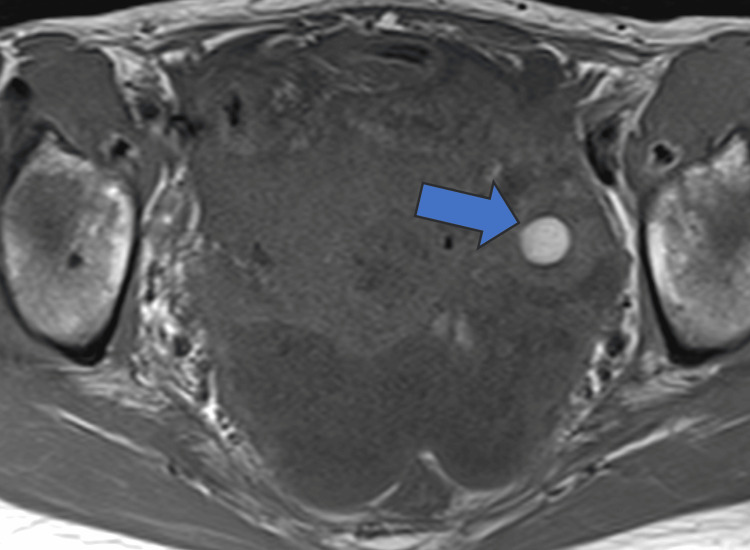
Gadolinium contrast-enhanced MRI scan (horizontal section) Hemorrhagic cyst of the left ovary (arrow).

After 14 h, all three blood cultures tested positive for Gram-positive Streptococcus. Although echocardiography was negative, three sets of blood cultures were positive, and the patient had a fever, The presence of granular casts was suggestive of possible glomerulonephritis, and a non-tender erythematous rash characteristic of Janeway lesions, and hence fulfilled one major and three minor criteria of the 2023 Duke International Society for Cardiovascular Infectious Diseases Criteria for IE, and therefore met the definition of a definite case of IE. With antibiotic treatment, the patient showed significant systemic improvement, particularly regarding her chest pain, diarrhea, and hematuria on day 2 following her admission. Despite the ongoing lower abdominal pain, we opted to persist with intravenous antibiotic treatment and symptom monitoring as IE was already established, and other health indicators were showing improvement. While hematuria did not align with typical IE symptoms, considering the patient's history of multiple symptom presentations, we directed our attention to improving parameters that led us to suspect that her urinary symptoms might be linked to an immunological phenomenon. Alongside these tests, the possible sites for the initial bacterial entry were also investigated. The patient underwent dermatological and orthodontic examinations, as skin tears and dental caries represent the most common sites of entry for Streptococcus; however, these revealed no significant anomalies. On day 5 after admission, the patient’s final blood culture results confirmed the presence of GAS, so the antibiotics were reduced to penicillin 400,0000 units q4h. However, on the same day the final blood culture results came in, the patient complained of worsening abdominal tenderness, of 8/10 on a subjective pain scale. Although her treatment was going well in one sense, her abdominal symptoms did not match the typical healing trajectory for GAS infections. We therefore revisited the medical interview process and openly asked the patient if she had noticed any other changes before and after the onset of her fever. She recalled that she had experienced an increase in vaginal discharge before her admission, and severe abdominal pain associated with her menstrual cycles, prompting a gynecological investigation. At this gynecological examination, she had complained of uterine and right-side adnexal tenderness. Although we were still in doubt regarding the relevance of these symptoms to her GAS infection, as this bacterium is highly unlikely to cause gynecological infections, we decided to perform pelvic magnetic resonance imaging (MRI). This revealed peritoneal thickening, fluid retention in the Douglas fossa, edema of the right fallopian tube, and a hemorrhagic cyst in the left ovary that was suggestive of endometriosis. A subsequent culdocentesis performed by a gynecologist on day 7 following her admission confirmed the presence of thick pus, leading us to suspect that the original source of the infection was, in fact, through the pelvic cavity (Figures 1A, 1B).

**Figure 2:**
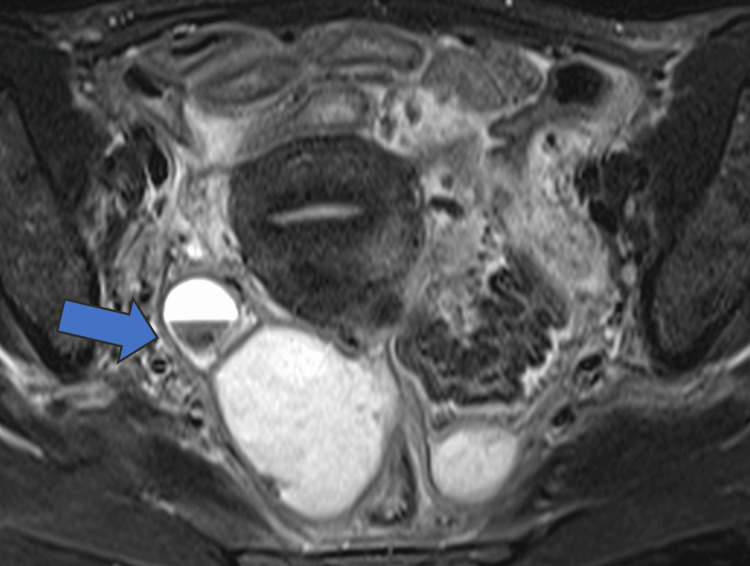
Gadolinium contrast-enhanced MRI scan (horizontal section) Right fallopian tube mass edema (arrow).

This prompted us to restart antibiotics targeting anaerobic bacteria with sulbactam/ampicillin (3 g q6h). The patient’s abdominal pain subsided after the culdocentesis, as well as a round of pus drainage. Although no culture grew from the extracted pus due to the antibiotic treatment the patient had just undergone, the pus at the Douglas fossa disappeared after 33 days of antibiotic treatment, with no further symptom recurrence (Figure 2).

## Discussion

Here, we reported a case of IE caused by GAS, where premature closure led to a delay in the assessment of her abdominal pain. The diagnosis of IE was made based on the patient's history, physical examination, and test results. However, despite the improvement of these factors, the abdominal pain persisted. This differed significantly from the healing trajectory of her other symptoms. This inconsistency prompted us to reassess our diagnosis. By openly questioning the patient such as “Do you have any idea what may have caused this symptom?”, we identified the original infected organ responsible for the IE. Ultimately, the patient was discharged with a favorable prognosis. This case brings forth two significant discussion points.

GAS infection of the gynecological system

We suspect that the patient in this case initially had endometriosis. This likely resulted in right fallopian tube edema due to hemoretention during menstruation. Consequently, the compromised tubes may have become susceptible to infection, leading to an inflammatory pelvic disease. This subsequently escalated to septicemia and IE. Her chest pain may have been caused by pleuritis and pleural effusion. Diarrhea may have been caused by systemic toxicity from the GAS or the spread of inflammation from the pelvis.

Mead et al. [9] studied the probability of GAS colonization in pregnant women pre-delivery. They cultured rectovaginal swabs at the time of GBS screening, and only found GAS colonization in 0.03% of the women, compared to GBS colonization of up to 20.1%. As the vagina is a rare reservoir for GAS, vaginal infection in adult women is infrequent. Only a few cases of this type of infection have been reported in the 23-41 age range [10]. Delahaye et al. [11] conducted a study on the entry sites of bacteria that led to IE. Their study revealed that the percentage of infections stemming from gynecological entry sites was as low as 10%. Based on these data, GAS infection can be considered a rare cause of vaginal infection, and even less likely to cause endocarditis, in premenopausal women. However, investigating the entry sites of bacteria that cause IE is essential for preventing recurrent infections. Had we overlooked the origin of the infection in this case and solely administered antibiotics for four weeks without draining the pus, the patient may have experienced a recurring infection. The critical question remains whether there were any apparent indications to suspect this minor route of infection in the first place.

The diagnostic process and the significance of patient perspectives

In this case, the patient presented with diverse symptoms. Nevertheless, we were able to swiftly diagnose IE by compiling information from interviews, physical examinations, and laboratory results. Previous studies have emphasized integrating various information types during diagnoses [12]. Medical interviews accounted for 80% of the diagnoses in that study, underscoring their pivotal role in the process [13]. However, one must be wary of cognitive biases, which can easily skew diagnoses [14]. In this instance, because the diagnosis of IE was promptly made using many different sources of information, likely, the causative origin of IE was not carefully considered during the initial consultation. Such an oversight can be attributed to satisfaction bias, a common occurrence in clinical settings [15]. In addition, the patient thought that the process of making the diagnosis was complete when she was diagnosed with IE and did not consider the possibility of additional diagnoses (premature closure). Another possible influence may have been the patient's tendency to disregard symptoms that did not fit the diagnosis of IE (e.g., abdominal pain), and to place importance only on information that was favorable to a diagnosis of IE, such as the heart murmur and Janeway lesions (compensation bias). To avoid these biases, the importance of physical examination should be stressed, or multiple cognitive reinforcement strategies should be used [16-18]. In this case, the key to the diagnosis was using open-ended questions posed directly to the patient.

It was previously reported that the intuitive diagnostic approach (system 1), the analytical diagnostic approach (system 2), and an open question posed by the physician (in this case, asking the patient’s perspective on the disease) proved to be very effective [19]. One study discusses the lateral approach (system 3), in which clinicians use open-ended questions to uncover patients' hidden concerns. This method requires careful consideration of anchoring bias but can be aided by using systems 1 and 2, as in our case [19]. Therefore, when diagnoses become challenging, it is imperative to engage patients in diagnostic processes to actively engage patients in diagnostic processes.

## Conclusions

We encountered a case of vaginal infection with GAS leading to IE. This case underscores the importance of reviewing the history when it does not fit the expected clinical course and the importance of posing open-ended questions to patients, such as system 3, when the diagnosis is unclear.
